# Prevalence and Determinants of Exclusive Breastfeeding Among Mothers of Children Aged 6–24 Months in the Aseer Region

**DOI:** 10.7759/cureus.66788

**Published:** 2024-08-13

**Authors:** Abdulelah M Abusabah, Hamza R Derkaoui, Hamad M Abusebah, Abdullah A Assiri, Norah H Assiry, Aidah S Al-Asmari, Fatima M Al Jaber, Halimah A Alshahrani, Eman M Alshahrani, Saada H Alshomrani, Aishah AlShahrani, Fatma A Alhubini, Khayria S Al-Ahmari, Layla A Alwalah, Tahani M Al-Madghidi, Alia I Al Asmi, Eman I Alzein, Mithheba A Assiry, Sana I Alzain, Mosiah I Assiri, Samirah A Al Asmari, Fatima I Al-Zein

**Affiliations:** 1 Preventive Medicine, King Khalid University, Abha, SAU; 2 Preventive Medicine, Ministry of Health, Abha, SAU; 3 Pediatric Intensive Care Unit, Khamis Mushayt Maternity and Children Hospital, Khamis Mushayt, SAU; 4 Family Medicine, Khamis Mushayt Health Sector, Khamis Mushayt, SAU; 5 Nursing, Ministry of Health, Abha, SAU; 6 Nursing, Ministry of Health, Khamis Mushayt, SAU; 7 Nursing, Ministry of Health Holdings, Khamis Mushayt, SAU; 8 Nursing, Ministry of Health, ​Ahad Rufaida, SAU; 9 Nursing, Ministry of Health, Rijal Almaa, SAU; 10 Nursing, Ministry of Health, Muhayil, SAU

**Keywords:** saudi arabia, perception, knowledge, determinants, prevalence, exclusive breastfeeding

## Abstract

Background

Exclusive breastfeeding (EBF) is crucial for infant and maternal health, providing optimal nutrition and immune protection for infants while reducing maternal postpartum depression and cancer risk. However, global trends show an early cessation of breastfeeding and the introduction of other foods. This study aims to determine the prevalence and factors influencing EBF among mothers in the Aseer region of Saudi Arabia.

Methodology

A cross-sectional study using a multistage cluster sampling approach was conducted from January to March 2024 in primary healthcare centers across the Aseer region. Participants included mothers of children aged 6-24 months attending primary healthcare centers for immunizations or routine check-ups. A pretested structured questionnaire, developed after an extensive literature review and expert consultation, was used to interview consenting participants. The questionnaire covered sociodemographic data, obstetric and medical history, child data, EBF practices, maternal knowledge and perception of breastfeeding, counseling about breastfeeding, antenatal care, breastfeeding support, and barriers and motivators of EBF.

Results

A total of 1,008 eligible mothers were included. Only 131 (13%) fulfilled the World Health Organization criteria for EBF. Moreover, 257 (25.5%) mothers initiated breastfeeding within the first hour after delivery, and 387 (38.4%) exclusively breastfed for six months or more. EBF was less frequent among mothers with higher education (8%, n = 2) compared to those with lower education (23%, n = 23, p = 0.017). EBF was also less frequent among mothers who delivered via cesarean section (7.9%, n = 28) compared to those who had a vaginal delivery (15.8%, n = 103, p = 0.001). Conversely, EBF was more common among mothers with more than five pregnancies (15.8%, n = 45) compared to those with one to two pregnancies (9.4%, n = 37, p = 0.023). EBF was also more common among mothers who had breastfed more than four children (16.7%, n = 39) compared to those who had not (12.1%, n = 49, p = 0.048). Finally, EBF was more common among mothers without postpartum complications (13.4%, n = 131) or whose infants had no birth complications (13.4%, n = 128) compared to their respective counterparts (p = 0.029 and p = 0.048, respectively).

Conclusions

This study found a low prevalence of EBF in the Aseer region, despite high maternal knowledge and positive perceptions. Factors such as low education, medical barriers, unemployment, and high parity were associated with increased EBF rates. Interventions should focus on improving workplace support and increasing maternal awareness of EBF recommendations.

## Introduction

Breastfeeding entails introducing breast milk into a baby’s diet [1]. It is a healthy approach to feeding a baby that has numerous advantages for both the mother and the child. Breast milk includes nutrients essential for the development of the brain and antibodies that help defend against infection and support the development of a baby’s immune system [2,3]. Additionally, there are benefits to exclusive nursing for mothers, such as weight loss following childbirth, a decreased risk of postpartum depression, and a decreased risk of ovarian and breast cancer [4,5]. Breast milk is a complete food that contains all the nutrients a baby needs for healthy growth and development. The most essential aspect of maternal and child health is breastfeeding, which provides nutritional and immunity benefits to both the mother and the infant [6,7].

The World Health Organization (WHO) recommends that babies be exclusively breastfed for the first six months of their lives [8,9]. This means that babies should drink only breast milk and no other liquids or foods. After the first six months, babies can start to eat solid foods, but they should continue to breastfeed until they are at least two years old [10]. The literature makes it clear that exclusive breastfeeding (EBF) is beneficial for the mother and the child in the short and long terms [10]. The advantages of EBF for the baby include decreased susceptibility to viral infections, consistent growth and cognitive development, and a lower chance of childhood obesity, diabetes mellitus, and hypertension [10,11]. The benefits of EBF for the mother include improved mother-child bonding and a decreased risk of type 2 diabetes, depression, postpartum blood loss, breast cancer, and ovarian cancer [12,13].

Notwithstanding these advantages, global trends indicate that the custom in many societies worldwide is the early discontinuation of nursing and the haphazard introduction of liquids, solids, and semi-solid meals [14,15]. Therefore, encouraging and supporting EBF at all levels of healthcare requires an understanding of these variables and an investigation of their relationships with breastfeeding practices. Accordingly, this study aims to identify the prevalence and determinants of EBF practices among mothers in the Aseer region of Saudi Arabia.

## Materials and methods

An analytical cross-sectional study was conducted in the Aseer region, targeting a representative sample of mothers with children aged 6-24 months who visited selected primary healthcare centers (PHCCs) to request immunizations or routine examinations for their children. The study was conducted from January to March 2024 in a PHCC in the Aseer region. Women with babies aged 6-24 months who lived in the Aseer region and agreed to participate in the study were included. Mothers whose children had any medical conditions that precluded EBF or did not complete the study survey were excluded. A multistage cluster sampling approach was employed to ensure adequate representation of participants from all regions within Aseer. In the first stage, a list of all health sectors within the region was compiled, and six sectors were randomly selected to serve as primary sampling units. In the second stage, within each selected sector, three PHCCs were selected by a simple random sample as secondary sampling units. Finally, from each of the three selected PHCCs within each sector, a list of all eligible participants was compiled. The required number of participants from each PHCC was then selected using simple random sampling.

The sample size was calculated using Epi Info software (Centers for Disease Control and Prevention, Atlanta, Georgia, USA) based on a prevalence rate of 26% for EBF practices [16]. With a precision of 3%, a confidence level of 95%, and a margin of error of 0.05, a minimum sample size of 830 mothers was determined. To account for potential non-response or incomplete surveys, the sample size was increased by 10%, resulting in a final target sample size of 913 mothers.

A comprehensive review of existing literature on EBF was conducted, accompanied by consultations with experts. Based on insights gained from the literature and expert consultations, a structured questionnaire was developed to capture a broad spectrum of factors related to EBF. The questionnaire addressed multiple domains, such as women’s sociodemographic data, obstetric and medical history, children’s data, EBF practices, maternal knowledge and perceptions of breastfeeding, breastfeeding counseling, antenatal care, breastfeeding support, and the barriers and motivators influencing EBF. To ensure accessibility for the target population in the Aseer region, the questionnaire was translated into Arabic. Subsequently, the face and content validity of the questionnaire were evaluated by a panel of four experts to ensure the clarity, relevance, and comprehensiveness of the questions. A pilot test was conducted on a sample of 35 mothers, who were excluded from the final study sample, to assess the questionnaire’s clarity, understandability, and practicality. This pilot testing phase facilitated the identification and resolution of any potential issues or ambiguities within the questionnaire. Furthermore, female healthcare workers, including nurses and doctors, who were responsible for administering the interviews, received training in data collection techniques and the use of the questionnaire during the pilot study. This training was essential to ensure consistency and accuracy in the data collection process.

The data were collected, reviewed, and then fed to SPSS version 26 (Released 2019; IBM Corp., Armonk, NY, USA). All statistical methods used were two-tailed with an alpha level of 0.05, with results considered significant if the p-value was less than or equal to 0.05. Descriptive analysis for categorical data was conducted using frequencies and percentages, whereas numerical data were presented in the form of means and standard deviations. All graphs were created using Microsoft Excel software (Microsoft Corp., Redmond, WA, USA). Cross-tabulation was used to show the factors associated with EBF and to assess the relationship between knowledge and attitude, using the Pearson chi-square test and the exact probability test for small frequency distributions. Multivariable logistic regression analysis was employed to identify independent predictors of EBF among the factors that showed a significant association (p < 0.05) in the bivariate analysis. The adjusted odds ratios (AORs) and their corresponding 95% confidence intervals were calculated to estimate the strength and significance of the associations after adjusting for all other factors in the model.

## Results

A total of 1,008 eligible mothers were included in the study. The mothers’ ages ranged from 18 to 48 years, with a mean age of 32.5 ± 6.1 years. Exactly 55.2% were university graduates, and 32.4% had a high school education. A total of 23.3% were employed, with salaries ranging from SR 5,000-10,000 among 26% to more than SR 10,000 among 37.9%. Only 81 (8%) respondents had chronic health problems, and 34 (3.4%) had medical problems that prevented them from feeding the baby.

**Figure 1:**
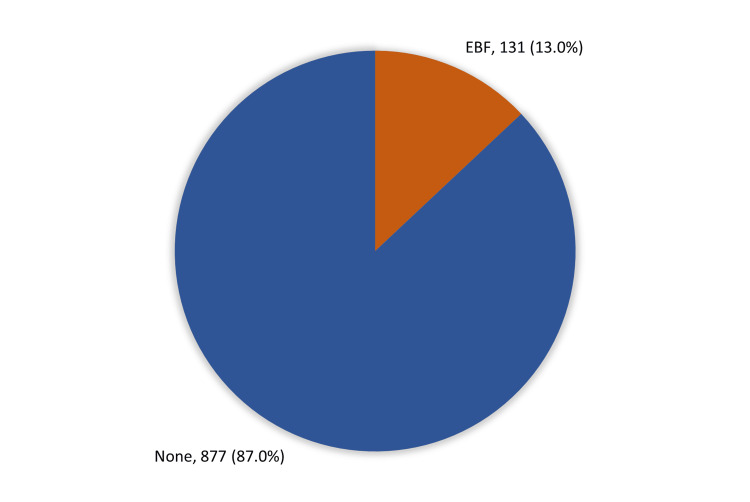
Prevalence of exclusive breastfeeding (EBF) among mothers of children aged 6-24 months in the Aseer region.

As for the prevalence of EBF (Figure 1), only 131 (13%) respondents fulfilled the WHO criteria for EBF; most of them (87%) did not. A total of 93 (9.2%) respondents did not breastfeed their babies, and only 257 (25.5%) breastfed their babies within one hour of delivery. EBF maintained for six months or more was observed among 387 (38.4%) respondents.

Table 1 shows the distribution of EBF by mothers’ bio-demographic data. A total of 23% of minimally educated mothers practiced EBF versus 8% of those with a postgraduate degree (p = 0.017). EBF was also reported by 14% of non-employed mothers compared to 9.8% of employed mothers (p = 0.047). Likewise, EBF was reported by 13.2% of mothers without medical problems preventing them from feeding their babies versus 5.9% of those with medical problems (p = 0.049).

**Table 1:** Distribution of exclusive breastfeeding by mothers’ bio-demographic data P: Pearson chi-square test; ^: exact probability test; * p < 0.05 (significant).

Bio-demographic data	Total		Exclusive breastfeeding				P-value
			Yes		No		
	N	%	N	%	N	%	
Mother’s age in years							0.840
18–24	96	9.5%	11	11.5%	85	88.5%	
25–29	212	21.0%	31	14.6%	181	85.4%	
30–35	304	30.2%	40	13.2%	264	86.8%	
>35	396	39.3%	49	12.4%	347	87.6%	
Marital status							0.837^
Married	980	97.2%	127	13.0%	853	87.0%	
Divorced/Separated	28	2.8%	4	14.3%	24	85.7%	
Educational level							0.017*
Below high school	100	9.9%	23	23.0%	77	77.0%	
High school	327	32.4%	40	12.2%	287	87.8%	
Bachelor’s degree	556	55.2%	66	11.9%	490	88.1%	
Postgraduate degree	25	2.5%	2	8.0%	23	92.0%	
Family income							0.353
SR <5,000	110	10.9%	14	12.7%	96	87.3%	
SR 5,000–10,000	399	39.6%	47	11.8%	352	88.2%	
SR 11,000–15,000	242	24.0%	36	14.9%	206	85.1%	
SR >15,000	119	11.8%	11	9.2%	108	90.8%	
I do not know	138	13.7%	23	16.7%	115	83.3%	
Employment							0.047*
Yes	235	23.3%	23	9.8%	212	90.2%	
No	773	76.7%	108	14.0%	665	86.0%	
Salary							0.179
SR <5,000	19	8.1%	4	21.1%	15	78.9%	
SR 5,000–10,000	61	26.0%	8	13.1%	53	86.9%	
SR >10,000	89	37.9%	7	7.9%	82	92.1%	
Not indicated	66	28.1%	4	6.1%	62	93.9%	
Mother’s chronic disease							0.612
Yes	81	8.0%	12	14.8%	69	85.2%	
No	927	92.0%	119	12.8%	808	87.2%	
Medical problems preventing mother from feeding the baby							0.049*^
Yes	34	3.4%	2	5.9%	32	94.1%	
No	974	96.6%	129	13.2%	845	86.8%	

Table 2 shows the distribution of EBF by mothers’ obstetric history. EBF was reported by 15.8% of grand multigravida women (more than five pregnancies) compared to 9.4% of those with one to two previous pregnancies (p = 0.023). EBF was also reported by 16.7% of women with more than four children versus 12.1% of mothers with no breastfed children (p = 0.048). Additionally, EBF was reported by 15.8% of women with normal vaginal delivery compared to 7.9% of those with cesarean section (p = 0.001).

**Table 2:** Distribution of exclusive breastfeeding by mothers’ obstetric history. P: Pearson chi-square test; ^: exact probability test; * p < 0.05 (significant).

Obstetric history	Total		Exclusive breastfeeding				P-value
			Yes		No		
	N	%	N	%	N	%	
Number of pregnancies							0.023*
1–2	394	39.1%	37	9.4%	357	90.6%	
3–4	329	32.6%	49	14.9%	280	85.1%	
5+	285	28.3%	45	15.8%	240	84.2%	
Number of breastfed children							0.048*
None	406	40.3%	49	12.1%	357	87.9%	
<4 children	369	36.6%	43	11.7%	326	88.3%	
>4 children	233	23.1%	39	16.7%	194	83.3%	
Duration before getting pregnant again after the last child							0.277
1–5 months	46	5.7%	9	19.6%	37	80.4%	
6–12 months	199	24.5%	22	11.1%	177	88.9%	
13–24 months	192	23.6%	24	12.5%	168	87.5%	
>24 months	376	46.2%	59	15.7%	317	84.3%	
Complications during previous pregnancies							0.466
Yes	69	6.8%	7	10.1%	62	89.9%	
No	939	93.2%	124	13.2%	815	86.8%	
Mode of delivery of the last child							0.001*
Cesarean section	355	35.2%	28	7.9%	327	92.1%	
Vaginal delivery	653	64.8%	103	15.8%	550	84.2%	
Type of health facility							0.616
Private health facility	217	21.5%	26	12.0%	191	88.0%	
Public health facility	791	78.5%	105	13.3%	686	86.7%	
Child’s age in months							0.133
6–11	508	50.4%	58	11.4%	450	88.6%	
12–24	500	49.6%	73	14.6%	427	85.4%	

Table 3 shows the factors associated with EBF among the women in the study. EBF was reported by 13.4% of mothers with no complications during the postpartum period versus none of those with complications (p = 0.029). EBF was also reported by 13.4% of mothers with the last child free of complications, compared to 5.8% of those with the last child having complications (p = 0.048).

**Table 3:** Factors associated with exclusive breastfeeding among the study women. P: Pearson chi-square test; ^: exact probability test; * p < 0.05 (significant).

Factors	Total		Exclusive breastfeeding				P-value
			Yes		No		
	N	%	N	%	N	%	
Birth weight of the last baby							0.189
<2.5 kg	278	27.9%	36	12.9%	242	87.1%	
2.5–3.5 kg	658	66.1%	78	11.9%	580	88.1%	
>3.5 kg	60	6.0%	12	20.0%	48	80.0%	
Mother’s complications during the last pregnancy							0.659
Yes	79	7.8%	9	11.4%	70	88.6%	
No	929	92.2%	122	13.1%	807	86.9%	
Mother’s complications during the postpartum period							0.029*^
Yes	31	3.1%	0	0.0%	31	100.0%	
No	977	96.9%	131	13.4%	846	86.6%	
Any mental health problems, such as postpartum depression							0.729
Yes	104	10.3%	10	9.6%	94	90.4%	
No	904	89.7%	121	13.4%	783	86.6%	
Last child at birth complications							0.048*
Yes	52	5.2%	3	5.8%	49	94.2%	
No	956	94.8%	128	13.4%	828	86.6%	
Number of postnatal visits attended							0.339
None	540	53.6%	62	11.5%	478	88.5%	
1	286	28.4%	40	14.0%	246	86.0%	
2	106	10.5%	14	13.2%	92	86.8%	
3	33	3.3%	6	18.2%	27	81.8%	
4 or more	43	4.3%	9	20.9%	34	79.1%	
Attended breastfeeding counseling							0.271
Yes	327	32.4%	48	14.7%	279	85.3%	
No	681	67.6%	83	12.2%	598	87.8%	
Number of breastfeeding counseling sessions attended							0.947^
1	198	60.6%	30	15.2%	168	84.8%	
2	82	25.1%	12	14.6%	70	85.4%	
3	25	7.6%	4	16.0%	21	84.0%	
4	8	2.4%	1	12.5%	7	87.5%	
5 or more	14	4.3%	1	7.1%	13	92.9%	
Received help with breastfeeding through verbal guidance or demonstrations while in the hospital for the delivery of the baby							0.558
Yes	478	47.4%	59	12.3%	419	87.7%	
No	530	52.6%	72	13.6%	458	86.4%	

**Figure 2:**
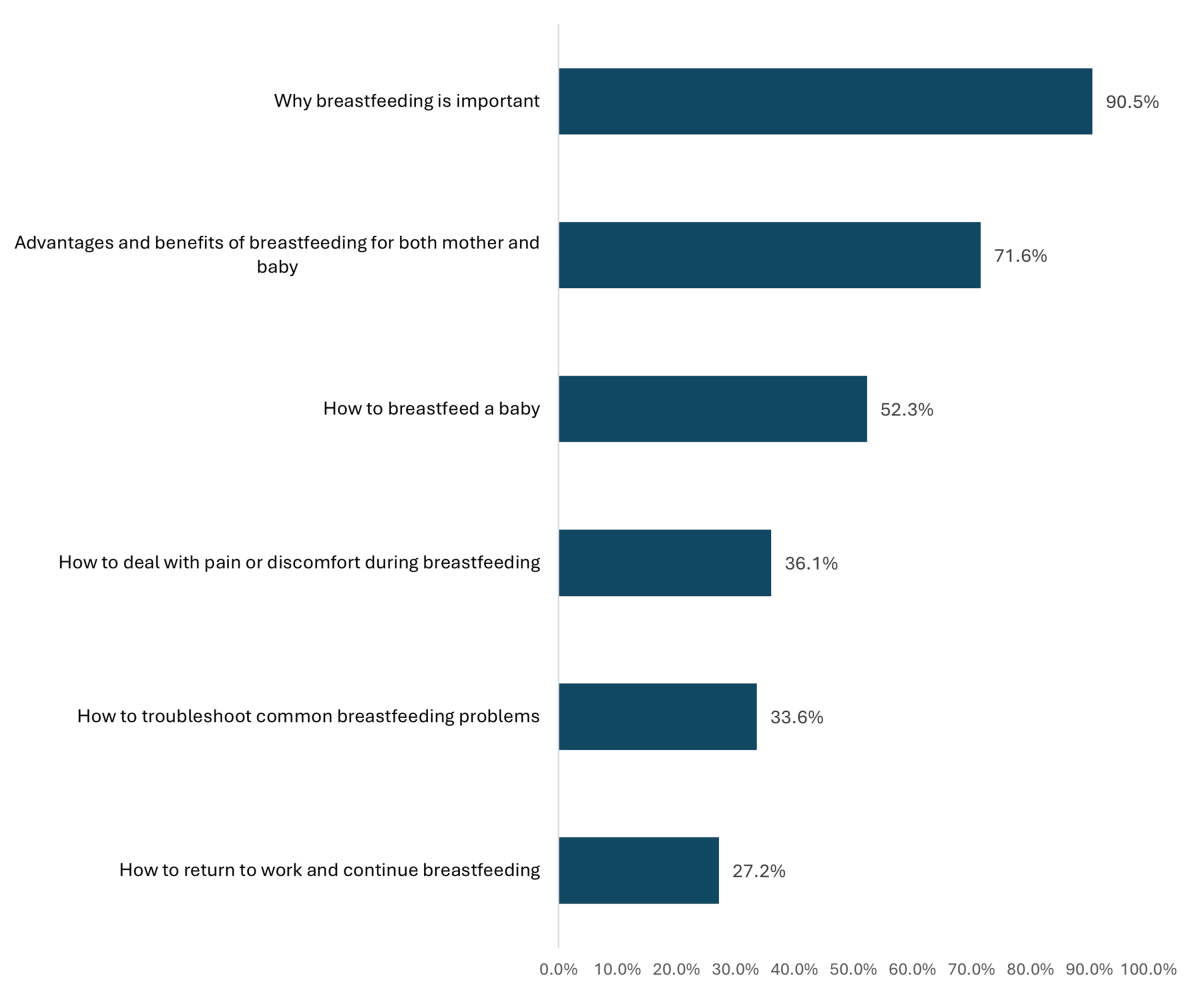
Issues discussed during breastfeeding counseling sessions with mothers (n = 327). The proportion of mothers who attended breastfeeding counseling sessions and reported that specific topics were discussed during those sessions. Mothers may report multiple topics.

Figure 2 shows the issues discussed during breastfeeding counseling sessions with mothers. The most reported items were why breastfeeding is important (90.5%), the advantages and benefits of breastfeeding for both the mother and the baby (71.6%), how to breastfeed a baby (52.3%), and how to deal with pain or discomfort during breastfeeding (36.1%).

Table 4 shows the distribution of EBF by mothers’ knowledge level. A total of 20.5% of mothers who correctly reported the best time to start breastfeeding had practiced EBF. Similarly, 24.3% of those who knew that EBF was important for the baby had also practiced EBF (p = 0.001).

**Table 4:** Distribution of exclusive breastfeeding by mothers’ knowledge level. P: Pearson chi-square test; ^: exact probability test; *: p < 0.05 (significant).

Knowledge about breastfeeding			Exclusive breastfeeding				P-value
	Total		Yes		No		
	N	%	N	%	N	%	
When is the best time to start breastfeeding?							0.001*^
Within the first hour of delivery	580	57.5%	119	20.5%	461	79.5%	
Within the first 24 hours of delivery	379	37.6%	11	2.9%	368	97.1%	
Within the first 3 days of delivery	32	3.2%	0	0.0%	32	100.0%	
Within the first week of delivery	17	1.7%	1	5.9%	16	94.1%	
How important is it to you to breastfeed your baby?							0.001*^
Extremely important	230	22.8%	56	24.3%	174	75.7%	
Very important	577	57.2%	69	12.0%	508	88.0%	
Somewhat important	169	16.8%	5	3.0%	164	97.0%	
Not important	24	2.4%	0	0.0%	24	100.0%	
Not important at all	8	0.8%	1	12.5%	7	87.5%	
Breastfeeding is not recommended for birth parents who have							0.391
HIV	639	63.4%	88	13.8%	551	86.2%	
Tuberculosis that has been treated	57	5.7%	10	17.5%	47	82.5%	
Hepatitis C virus	282	28.0%	30	10.6%	252	89.4%	
Diabetes mellitus	30	3.0%	3	10.0%	27	90.0%	
A mother who is breastfeeding exclusively is most likely to							0.204^
Have a lower risk of postpartum depression	722	71.6%	93	12.9%	629	87.1%	
Lose weight more quickly after childbirth	892	88.5%	119	13.3%	773	86.7%	
Have a lower risk of breast cancer	816	81.0%	112	13.7%	704	86.3%	
Have a lower risk of ovarian cancer	719	71.3%	103	14.3%	616	85.7%	
All of the above	3	0.3%	0	0.0%	3	100.0%	

Table 5 shows the attitudes and perceptions of women in the study toward breastfeeding children. Exactly 55.4% of the women reported that all the items listed, i.e., feeling confident in their ability to breastfeed, learning more about the benefits of EBF, having a strong support system, and having access to lactation support, made them more likely to breastfeed exclusively. Moreover, 49.4% of respondents reported concerns about milk supply as the primary factor making them less likely to breastfeed exclusively. Exactly 84.4% of the women reported a high likelihood of breastfeeding their babies, 53.1% believed that their baby would get sick if not breastfed exclusively, 33.3% thought it was serious if not breastfeed exclusively, and 96% believed it is beneficial to breastfeed one’s baby exclusively.

**Table 5:** Study women’s attitudes and perceptions toward breastfeeding children.

Attitude	N	%
What would make you more likely to breastfeed exclusively?		
Feeling confident in my ability to breastfeed	118	11.7%
Learning more about the benefits of exclusive breastfeeding	155	15.4%
Having a strong support system	137	13.6%
Having access to lactation support	40	4.0%
All of the above	558	55.4%
What would make you less likely to breastfeed exclusively?		
Having concerns about my milk supply	498	49.4%
Not having enough time to breastfeed	131	13.0%
Having to return to work early	89	8.8%
Feeling pressure from others to formula feed	45	4.5%
All of the above	245	24.3%
How likely are you to breastfeed your baby?		
Neither likely nor unlikely	41	4.1%
Somewhat likely	381	37.8%
Somewhat unlikely	81	8.0%
Very likely	470	46.6%
Very unlikely	35	3.5%
How likely do you think it is that your baby will get sick if you do not breastfeed exclusively?		
Not at all likely	256	25.4%
Not very likely	216	21.4%
Somewhat likely	335	33.2%
Very likely	201	19.9%
How serious do you think it is for your baby to get sick if you do not breastfeed exclusively?		
Not at all serious	274	27.2%
Not very serious	398	39.5%
Somewhat serious	252	25.0%
Very serious	84	8.3%
How beneficial do you think it is to breastfeed your baby exclusively?		
Not at all beneficial	8	0.8%
Not very beneficial	32	3.2%
Somewhat beneficial	121	12.0%
Very beneficial	847	84.0%

The model showed that among all included univariate significant predictors, only high education (OR = 0.82) and cesarean section as the mode of delivery (OR = 0.49) were significantly associated with a lower likelihood of EBF (18% and 51%, respectively) after adjusting for all other factors. Conversely, a higher number of pregnancies (OR = 1.11), knowing the best time to start breastfeeding (OR = 6.07), and understanding the importance of breastfeeding (OR = 2.34) were associated with a higher likelihood of EBF (11%, 6 times more, and 2.3 times more, respectively) (Table 6).

**Table 6:** Multiple logistic regression model for the predictors and determinants of exclusive breastfeeding among mothers in the Aseer region. AOR: adjusted odds ratio; CI: confidence interval; *: p < 0.05 (significant).

Factors	P-value	AOR	95% CI	
			Lower	Upper
High education	0.036*	0.82	1.01	2.36
Employment	0.342	1.31	0.75	2.30
Medical problems prevent mothers from feeding the baby	0.471	0.57	0.13	2.60
Number of pregnancies	0.049*	1.11	1.01	1.89
Number of breastfed children	0.371	1.14	0.86	1.50
Cesarean section as the mode of delivery	0.003*	0.49	0.31	0.79
Mother’s complications during the postpartum period	0.998	0.23	0.01	0.59
Last child at birth complications	0.778	1.14	0.47	2.78
Knowing the best time to start breastfeeding	0.001*	6.07	3.41	10.79
Knowing the importance of breastfeeding	0.001*	2.34	1.70	3.20

## Discussion

This study aimed to assess the prevalence and determinants of EBF among mothers in the Aseer region. The study found that only 13% fulfilled the WHO criteria for EBF. Several studies have been conducted on EBF among Saudi women. A study conducted in 2019 found that only 17% of Saudi women exclusively breastfeed their babies for the first six months of life, which is higher than the current study’s finding [17]. A similar finding in Saudi Arabia was made by Alyousefi et al. [18], who found that only 13.7% of all infants were exclusively breastfed at the age of six months. A higher rate of EBF was detected in Al-Ahsa by Ahmed and Salih, who found that 26% of babies were exclusively breastfed [16]. Recently, according to a local survey, 80.8% of Saudi Arabian newborns under the age of six months were not exclusively breastfed [19]. A relatively recent improvement to an EBF rate of 31.4% was reported [20], while only 8.3% of Saudi female teachers were reported to exclusively breastfeed in a previous study [21]. This wildly concerning downward trend in EBF reflects the significant variation in data between Saudi Arabian areas and time periods, which complicates analysis [22]. Although our study’s finding of 13% EBF prevalence is nearly identical to the 17% rate reported in Nigeria [23] and the 12.5% rate in Egypt [24], it is evident that our findings pale in comparison to those of studies conducted in Zanzibar (20.8%) [25], Ghana (38%) [26], Bangladesh (36%) [27], and Tanzania (24.1%) [28].

In reference to the current study, breastfeeding initiation within the first hour following delivery among 25.5% of respondents is a significant finding. This is far lower than both the previously discovered 65% rate in Nepal [29] and the WHO’s 100% aim [30]. The extremely low incidence of first-hour breastfeeding initiation found in this study may help to explain the low rates of EBF in the Aseer region as it has been shown to have an impact on future EBF practices [31]. Despite the established health benefits of skin-to-skin contact and early nursing for newborns, very little study has been done in the Middle East on these topics [32].

Regarding factors associated with EBF, this study showed that low education, unemployment, lack of child medical problems, higher pregnancies (more experience), previous breastfeeding of children, normal vaginal delivery, lack of mother complications during the postpartum period, and good knowledge among mothers about breastfeeding and its benefits were significantly associated with a higher prevalence of EBF. Other similar findings were reported by Alsulaimani [17], who found that having three to six children and a one-year interpregnancy interval were strong predictors of EBF. In contrast to the current study, Alzaheb et al. [20] found that Saudi nationals and babies born via cesarean delivery or at low birth weights were associated with a lower EBF rate. Conversely, the mother’s awareness of the recommended EBF duration was positively associated with EBF, which is consistent with the current study findings. However, the study also found that counseling about EBF and the number of antenatal care visits were not significantly associated with EBF practices. This suggests that simply providing information and healthcare visits may not be sufficient to promote EBF. The quality and effectiveness of breastfeeding counseling and the integration of breastfeeding support into routine antenatal and postnatal care may require further evaluation and improvement. Other studies examined various common factors, and several of these were associated with EBF (e.g., breastfeeding initiation, residence, and employment status). However, they disagreed on some of the other factors (such as the mother’s age, educational status, number of children, and family income) and reported conflicting associations with EBF in relation to them [33,34].

The discrepancies between the current study’s findings and those of other studies may be attributed to differences in study design, sampling methods, and data collection tools. Additionally, the unique cultural and socioeconomic context of the Aseer region, coupled with the healthcare system’s capacity to provide breastfeeding support, may contribute to the observed variations. These factors highlight the complexity of breastfeeding behaviors and the need for context-specific strategies to promote and support EBF.

The current study underscores the ongoing challenges in providing comprehensive breastfeeding support within the Aseer region, even with improvements in healthcare infrastructure. The limited availability of lactation consultants and specialized clinics, particularly in rural areas, creates barriers for mothers seeking adequate guidance. This highlights the need to integrate breastfeeding promotion and support into standard antenatal and postnatal care, especially in underserved communities. The study’s findings also emphasize the role of socioeconomic factors, revealing a counterintuitive association between lower education levels and increased EBF rates, potentially attributable to the influence of traditional beliefs and practices. This underscores the importance of culturally sensitive interventions that acknowledge existing knowledge while advocating for optimal breastfeeding practices.

The study was subject to some limitations. Of the limitations, the cross-sectional research design is the most crucial as it makes it impossible to infer causal relationships from the correlations found between significant variables and EBF practices. Second, because data on EBF rely on mothers’ memories of the time after giving birth, and some women might find it difficult to recall precisely when they started adding complementary foods or liquids to their child’s diet, this design is also susceptible to recall bias. The study’s relatively brief data collection period, spanning from January to March 2024, may not have adequately accounted for the potential impact of seasonal fluctuations or other temporal factors that could influence breastfeeding practices.

Further research is warranted to explore these influences in greater depth, particularly focusing on the interplay between cultural norms, socioeconomic factors, and healthcare support. Understanding these dynamics can help identify effective strategies to promote and support EBF in diverse settings, ultimately improving infant health outcomes.

## Conclusions

This study reveals an alarmingly low prevalence of EBF among mothers in the Aseer region, despite their reported high level of knowledge and positive perception of breastfeeding. The study identified several factors associated with higher EBF rates, including lower maternal education, unemployment, absence of medical barriers, and high parity. Notably, the study also found that early breastfeeding initiation rates were significantly lower than WHO recommendations. These findings underscore the urgent need for targeted interventions to improve EBF rates in the region. Such interventions should focus on enhancing early breastfeeding initiation through improved training and protocols, expanding access to lactation support services, particularly in rural areas, and improving the quality of breastfeeding counseling. Future initiatives must also consider the unique socioeconomic and cultural context of the region to ensure their effectiveness. Longitudinal research is recommended to gain a deeper understanding of the determinants of EBF and address the limitations inherent in cross-sectional studies. By implementing these strategies, we can strive to improve EBF rates and, consequently, infant health outcomes in the Aseer region and similar settings.

## Appendices

**Table 7:** Exclusive breastfeeding (EBF) questionnaire in English.

Demographic Information
Mother’s age:
……………. years
Marital status:
Married
Divorced
Widowed
Separated
Education level:
Illiterate
Primary school
Intermediate school
High school graduate
Bachelor’s degree
Master’s degree
Doctorate degree
Family income:
Class 1 <5,000 SAR
Class 2 >5,000–10,000 SAR
Class 3 >10,000–15,000 SAR
Class 4 >15,000 SAR
Employed
Yes
If yes what is your current salary…………………… SAR
No
Medical history
Have you ever been diagnosed with any chronic disease:
Yes
If yes, what are ………………………
No
Did you have any medical problems that prevented you from feeding your baby:
Yes, specify…………………………
No
Pregnancy history (M = male) (F = female)
Number of pregnancies
………………………………
Number of live births
M………………… F……………
Number of miscarriages
M…………………… F…………
Number of children who were breastfed:
M…………………… F…………
How long did you wait before getting pregnant again after your last child?
………………………………. Months
Any complications during previous pregnancies
………………………………
Mode of delivery of the last child:
Vaginal delivery
Cesarean section
Type of health facility:
Public health facility
Private health facility
Last baby’s age: ……………. Months
Last baby’s gender
Male
Female
Birth weight of the last baby:………….. gram
Any mother complications during the last pregnancy:
Yes …………….
No
Any mother complications during the postpartum period
Yes………….
No
Any mental health problems, such as postpartum depression
Yes ………….
No
Did your last child have any complications at birth:
Yes
If yes, what was it……………
No
How many antenatal clinics have you attended:
…………………..
How many postnatal clinics have you attended:
…………………..
Have you attended any breastfeeding counseling?
Yes
No…if no, go to question 28.
How many times have you attended breastfeeding counseling?
…………………………
Which of the following was discussed during breastfeeding counseling?
Why breastfeeding is important
Advantages and benefits of breastfeeding for both the mother and the baby
How to breastfeed a baby, including positioning and attachment
How to deal with pain or discomfort during breastfeeding
How to return to work and continue breastfeeding.
How to troubleshoot common breastfeeding problems
While you were in the hospital for the delivery of this baby, did anyone help you with breastfeeding by talking to you or showing you how to breastfeed your baby?
Yes
No
How long after your baby was born did you start breastfeeding?
During the first hour of delivery
2–24 hours
>24 hours
Not breastfed at all
How long did you exclusively breastfeed your baby?
………………Months
If you stop breastfeed your baby what were your reasons for stopping breastfeeding?
Not producing enough milk
Going back to work
Having pain while breastfeeding
A medical condition that made it difficult to breastfeed
Pressure by family or friends to stop breastfeeding
Other (please specify):……………….
Mother’s knowledge of EBF
When is the best time to start breastfeeding:
Within the first hour of delivery
Within the first 24 hours of delivery
Within the first 3 days of delivery
Within the first week of delivery
How important is it to you to breastfeed your baby?
Not important at all
Not very important
Somewhat important
Very important
Extremely important
Breastfeeding is not recommended for birth parents who have:
Hepatitis C
Tuberculosis that has been treated
Diabetes
HIV
Mother’s attitude toward EBF
A mother who is breastfeeding exclusively is most likely to:
Have a lower risk of postpartum depression
Lose weight more quickly after childbirth
Have a lower risk of breast cancer
Have a lower risk of ovarian cancer
All of the above
What would make you more likely to breastfeed exclusively?
Having a strong support system
Learning more about the benefits of EBF
Having access to lactation support
Feeling confident in my ability to breastfeed
All of the above
What would make you less likely to breastfeed exclusively?
Having to return to work early
Having concerns about my milk supply
Feeling pressure from others to formula feed
Not having enough time to breastfeed
All of the above
How likely are you to breastfeed your baby?
Very unlikely
Somewhat unlikely
Neither likely nor unlikely
Somewhat likely
Very likely
Mother’s practice toward EBF
Have you breastfed your last child?
Yes
No
How frequently did you breastfeed your last child?
On-demand
Regularly
Randomly
Perceived susceptibility:
How likely do you think it is that your baby will get sick if you do not breastfeed exclusively?
Very likely
Somewhat likely
Not very likely
Not at all likely
Perceived seriousness:
How serious do you think it is for your baby to get sick if you do not breastfeed exclusively?
Very serious
Somewhat serious
Not very serious
Not at all serious
Perceived benefits:
How beneficial do you think it is to breastfeed your baby exclusively?
Very beneficial
Somewhat beneficial
Not very beneficial
Not at all beneficial
Perceived barriers:
What are the barriers that you think might prevent you from breastfeeding your baby exclusively?
I don't have enough time to breastfeed
I'm not sure how to breastfeed
I'm worried about my milk supply
I'm worried about my baby getting sick
Other (please specify):
Cues to action:
What would motivate you to breastfeed your baby exclusively?
My doctor told me it’s the best thing for my baby
I want to give my baby the best start in life
I want to save money on formula
I want to bond with my baby
Other (please specify):
